# Comparison of inbred mouse strains shows diverse phenotypic outcomes of intervertebral disc aging

**DOI:** 10.1111/acel.13148

**Published:** 2020-04-22

**Authors:** Emanuel J. Novais, Victoria A. Tran, Jingya Miao, Katie Slaver, Andrew Sinensky, Nathaniel A. Dyment, Sankar Addya, Flora Szeri, Koen van de Wetering, Irving M. Shapiro, Makarand V. Risbud

**Affiliations:** ^1^ Department of Orthopedic Surgery Sidney Kimmel Medical College Philadelphia PA USA; ^2^ Graduate Program in Cell Biology and Regenerative Medicine Thomas Jefferson University Philadelphia PA USA; ^3^ Life and Health Sciences Research Institute (ICVS) School of Medicine University of Minho Braga Portugal; ^4^ ICVS/3B’s – PT Government Associate Laboratory Braga Portugal; ^5^ Department of Orthopaedic Surgery University of Pennsylvania Philadelphia PA USA; ^6^ Sidney Kimmel Cancer Center Thomas Jefferson University Philadelphia PA USA; ^7^ Department of Dermatology and Cutaneous Biology Sidney Kimmel Medical College Thomas Jefferson University Philadelphia PA USA; ^8^ The PXE International Center of Excellence in Research and Clinical Care Thomas Jefferson University Philadelphia PA USA; ^9^ The Jefferson Institute of Molecular Medicine Thomas Jefferson University Philadelphia PA USA

**Keywords:** Aging, calcification, intervertebral disc, LG/J, SM/J, transcriptome

## Abstract

Intervertebral disc degeneration presents a wide spectrum of clinically degenerative disc phenotypes; however, the contribution of genetic background to the degenerative outcomes has not been established. We characterized the spinal phenotype of 3 mouse strains with varying cartilage‐regenerative potential at 6 and 23 months: C57BL/6, LG/J and SM/J. All strains showed different aging phenotypes. Importantly, LG/J mice showed an increased prevalence of dystrophic disc calcification in caudal discs with aging. Quantitative‐histological analyses of LG/J and SM/J caudal discs evidenced accelerated degeneration compared to BL6, with cellular disorganization and cell loss together with fibrosis of the NP, respectively. Along with the higher grades of disc degeneration, SM/J, at 6M, also differed the most in terms of NP gene expression compared to other strains. Moreover, although we found common DEGs between BL6 and LG/J aging, most of them were divergent between the strains. Noteworthy, the common DEGs altered in both LG/J and BL6 aging were associated with inflammatory processes, response to stress, cell differentiation, cell metabolism and cell division. Results suggested that disc calcification in LG/J resulted from a dystrophic calcification process likely aggravated by cell death, matrix remodelling, changes in calcium/phosphate homeostasis and cell transformation. Lastly, we report 7 distinct phenotypes of human disc degeneration based on transcriptomic profiles, that presented similar pathways and DEGs found in aging mouse strains. Together, our results suggest that disc aging and degeneration depends on the genetic background and involves changes in various molecular pathways, which might help to explain the diverse phenotypes seen during disc disease.

## Graphical abstract

This study investigates how genetic background influences the acquisition of specific morphological phenotypes in the intervertebral disc with aging. By uncovering the aging transcriptomic signature of three inbred mouse strains, and distinct transcriptomic profiles in human degenerated discs, our studies shed light on the wide spectrum of phenotypes presented during disc disease.

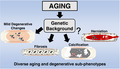

## 1 INTRODUCTION

Despite the multifactorial aetiology of disc degeneration, one of the critical risk factors is aging (Chanchairujira et al., 2007; Cheung et al., 2009). This is becoming more important as increasing lifespan is contributing to a rise in age‐dependent diseases. It is known that over the course of lifespan, discs experience decreased levels and quality of extracellular matrix proteins, compromised biomechanical properties, increase in inflammatory cytokine expression, catabolic processes and cell death (Caldeira et al., 2017; Ohnishi, Sudo, Tsujimoto, & Iwasaki, 2018). Similarly, senescence, herniations and calcifications have been correlated with degenerative phenotypes in the disc (Novais, Diekman, Shapiro, & Risbud, 2019). Accordingly, the term degenerative disc disease is an umbrella term that encompasses multiple phenotypes. It is therefore important to delineate the molecular basis of these distinct pathologies and their contribution to disc aging and degeneration.

The absence of an appropriate animal model is a barrier for a better understanding of the nuances of disc aging and degeneration. Many experimental models, ranging from large to small animals, and from mechanically induced injury to genetic manipulations have been developed for this purpose (Caldeira et al., 2017; Choi et al., 2018; Novais et al., 2019; Yang, Leung, Luk, Chan, & Cheung, 2009). Injury models rely on acute trauma, usually by annular puncture with consequent inflammatory and immune responses. While injury models are of interest to study acute herniations and the role of inflammation in pathology, they do not recapitulate the chronic cases of human disc degeneration, especially those related to aging (Yang et al., 2009). Likewise, genetic modification allows insights into how a single gene or protein contributes to the inflammation‐driven disc herniations and/or degeneration but does not capture the multivariable complexity of the aging phenotype (Gorth, Ottone, Shapiro, & Risbud, 2019). Nevertheless, models such as mice and rats are attractive for studying disc aging due to their genetic similarity with humans and the availability of tools for population genetics. Importantly, several studies have shown comparable changes in histology, mechanical properties, cellular responses and matrix remodelling during disc degeneration in mice and humans (Choi et al., 2018; Ohnishi et al., 2018).

SM/J, a poor healer mouse strain, and LG/J, a super healer strain, have recently become an attractive system to study disc degeneration due to their opposing phenotypes (Choi et al., 2018) While SM/J mice showed early‐onset, spontaneous disc degeneration, it is not known how their genetic background influences disc aging. To address these questions, we characterized the spinal phenotype of 6‐month‐old (6M) and 23‐month‐old (23M) SM/J, LG/J and C57BL/6 (BL6) mice, the most widely used strain in aging and disc research which exhibits poor healing capabilities compared to LG/J mice (Choi et al., 2018). Our results show that LG/J mice are prone to developing dystrophic disc calcification in caudal spine with aging and the aging phenotype is distinct between 3 strains. Transcriptomic analysis showed SM/J differs the most from the other strains at 6M, exhibiting highest grades of degeneration. Interestingly, during aging, strains shared only a small subset of genes related to stress response, cell differentiation, inflammation‐related pathways and cell metabolism with LG/J mice showing differences in pathways concerned with cell death, calcium homeostasis and endochondral processes. Signatures of calcium homeostasis, cell death and endochondral genes were also captured in hierarchical clustering analysis of transcriptomic data from human NP tissues. Together, the findings highlight the contribution of the genetic background to spectrum of degenerative phenotypes seen during aging and disc degeneration.

## 2 RESULTS

### 2.1 LG/J mice have a higher prevalence of age‐dependent disc calcification than SM/J and BL6

**FIGURE 1:**
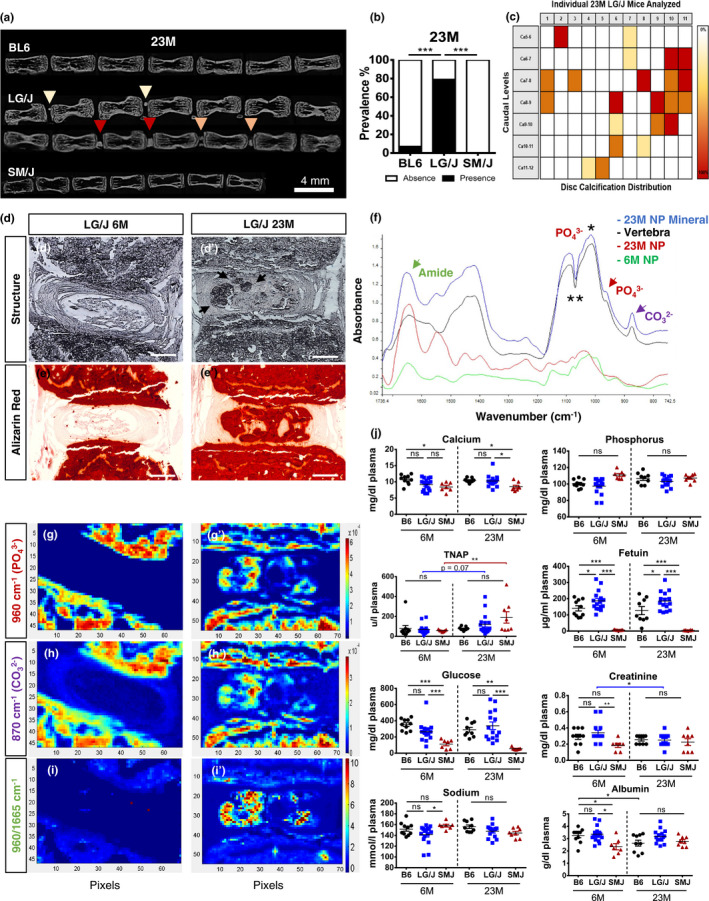
LG/J mice present high prevalence of age‐dependent disc calcification. (a‐c) μCT showed higher prevalence and widespread distribution of disc calcification in caudal spine of 23M LG/J mice. Yellow, orange and red arrows (a) and colour scale (c) represent small, medium and large size calcifications, respectively. *p* ≤ .001***, χ^2^ test, BL6 (*n* = 10), LG/J (*n* = 14) and SM/J (*n* = 8). (d‐d’) Disc calcifications in LG/J mice were present in both NP and AF. (e‐e’) Alizarin Red staining showed staining of the disc, not limited to the calcified nodules. (f) FTIR analysis showed comparable spectral profile between disc mineralization and vertebrae. Green arrow—amide peak – 1,665/cm; red arrow—phosphate peak – 960/cm; purple arrow—carbonate peak – 870/cm; *phosphate peak—1,030/cm; ** tape artefact. (g‐h’) Phosphate and carbonate levels in the disc were comparable to the vertebrae. (i‐i’) Phosphate to protein ratio was higher in the disc. (j) Plasma measurements of calcium, phosphorous, TNAP, fetuin, glucose, creatine, sodium and albumin from 6M and 23M mice. Mann–Whitney test was used for comparing differences between the groups. ns = not significant; *p* ≤ .05*; *p* ≤ .01**; *p* ≤ .01**; BL6 (*n* = 10), LG/J (*n* = 14) and SM/J (*n* = 8). Scale bar d‐e’ = 200 µm

To investigate how genetic background affects disc phenotype with aging, we analysed the spines of 6M and 23M BL6, LG/J and SM/J mice by μCT and histology. μCT analysis showed high prevalence of disc calcification in caudal spine of 23M LG/J mice with a comparable mineral density to vertebral cortical bone (Figure 1a‐c). Calcification presented a wide distribution, size and morphology (Figure 1c) and affected both NP and AF (Figure 1d‐e’). Noteworthy, several LG/J animals showed increased incidence of calcification at Ca6‐7, Ca7‐8 and Ca8‐9, whether this is due to differential loading at these levels remains to be seen. There was a lack of disc calcification in 23M‐old BL6 and SM/J mice (Figure 1a, Figure S2a) and in LG/J lumbar spine (Figure S2a). Alizarin Red staining showed presence of pronounced free calcium in 23M LG/J disc that was not restricted to the mineral nodules (Figure 1d‐e’ and Figure S2b). FTIR‐studies showed that this calcification was apatitic with a comparable spectral profile to the mineral in the vertebrae (Figure 1f). We analysed the peaks corresponding to phosphate—960/cm (Figure 1g‐g’), carbonate—870/cm (Figure 1h‐h’) and protein—1,665 cm^−1^ (Figure 1i‐i’). Although, phosphate and carbonate levels in the disc were comparable to the vertebrae (Figure 1g‐h’), the ratio of phosphate to protein content was much higher in the disc (Figure 1i‐i’). This suggested that LG/J disc mineralization was not associated with matrix and thus dystrophic in nature. While plasma analysis showed that 23M LG/J mice had a slight increase in levels of free calcium compared to SM/J, levels were comparable to BL6. With aging, both LG/J and SM/J mice showed increased activity of TNAP, without differences between the strains. Levels of fetuin‐A were higher in LG/J but virtually undetectable in the SM/J mice. Plasma glucose was significantly decreased in SM/J mice as compared to BL6 and LG/J mice without any strain‐dependent change in creatine, sodium and albumin (Figure 1j). These results suggested that there was no clear correlation between the plasma chemistry and strain‐specific mineralization phenotype. We then decided to focus on analysing caudal disc aging due to this calcification phenotype and to avoid confounding contribution of axial loading on lumbar spine associated with variations in body sizes between three strains (Espinoza Orías, Malhotra, & Elliott, 2009).

### 2.2 LG/J and SM/J mice have distinct phenotypes of age‐related disc degeneration compared to BL6

**FIGURE 2:**
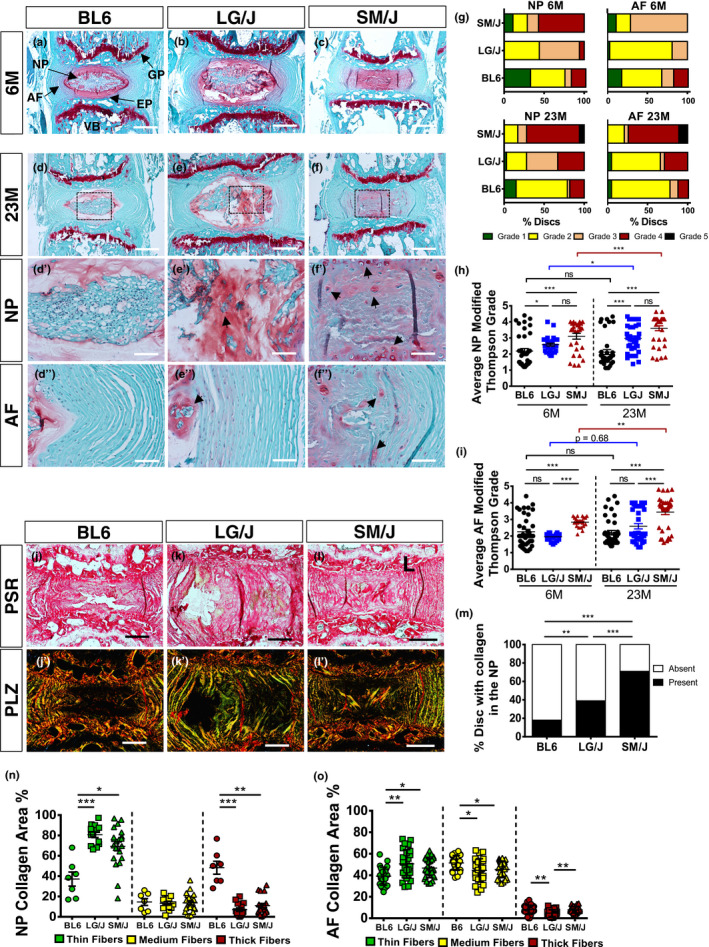
Inbred strains show distinct disc phenotypes during aging. (a‐f’’) Histology of 6M and 23M mice showed differences in tissue architecture and cell morphology in the NP, AF and EP. Panels d', e' and f' show the area enclosed in dotted boxes in panels d, e and f, respectively. (g‐i) Modified Thompson Grading distribution and averages showed small degenerative changes in LG/J mice and BL6 but high degenerative grades in SM/J mice at 6M. At 23M, both LG/J and SM/J mice showed higher degenerative grades than BL6 mice in the NP. (j‐m) Picro‐Sirius Red staining and polarized microscopy of discs (n‐o) Analysis of percentage of thin (green), intermediate (yellow) and thick fibres (red). Mann–Whitney test was used for comparing 6M to 23M in each strain. Kruskal–Wallis and chi‐squared test were used to perform comparison between the 3 strains. BL6 (*n* = 10), LG/J (*n* = 14) and SM/J (*n* = 8), 4 levels per strain were analysed. Scale bar A‐F and J‐L’= 200 µm; Scale bar d’‐f = 50 µm. AF, annulus fibrosus; EP, endplate; GP, growth plate; NP, nucleus pulposus; VB, vertebra

We performed histological analysis of caudal disc of 6M and 23M BL6, LG/J and SM/J mice. There were differences in tissue architecture and cell morphology in the NP, AF and EP compartments of all strains (Figure 2a‐i and Figure S2b). Starting at 6M, there were small degenerative changes in LG/J discs compared to BL6; notably, NP cell band showed more interspersed matrix (Figure 2a‐b). SM/J mice, on the other hand, showed a loss of NP/AF demarcation, a fewer NP cells, and presence of clefts (Figure 2c). At 23M, in contrast to LG/J and SM/J, BL6 mice showed very little changes in caudal spine associated with aging; disc compartments maintained their cellularity and demarcation (Figure 2d‐f’’). The aging phenotype of LG/J mice was characterized by an asymmetric cellular organization, with a healthier looking cell band on one side and a honeycomb organization on the other, paired with increased mucinous material and clefts in the inner AF (Figure 2e‐e’’). The SM/J phenotype was underscored by lack of cells in the NP, a clear increase in hypertrophic chondrocyte‐like cells in the endplates, and clefts throughout the disc (Figure 2f‐f’’). Histological changes were quantified using the modified Thompson Grading (Figure S3a) and evidenced different distribution and higher average grades of NP degeneration in LG/J and SM/J compared to BL6 mice at both 6M and 23M (Figure 2g,h). SM/J mice presented higher average AF grades compared to the other strains at both the time points (Figure 2g,i). Picro‐Sirius Red staining and polarized microscopy showed increased collagen staining in the NP of LG/J and SM/J mice than BL6 (Figure 2j‐m) and a similar increase in thin fibres, along with a decrease in thick fibres in LG/J and SM/J NP and AF compartments compared to BL6 mice (Figure 2j‐l’,n,o).

### 2.3 Aged inbred mice strains evidenced changes in extracellular matrix composition

**FIGURE 3:**
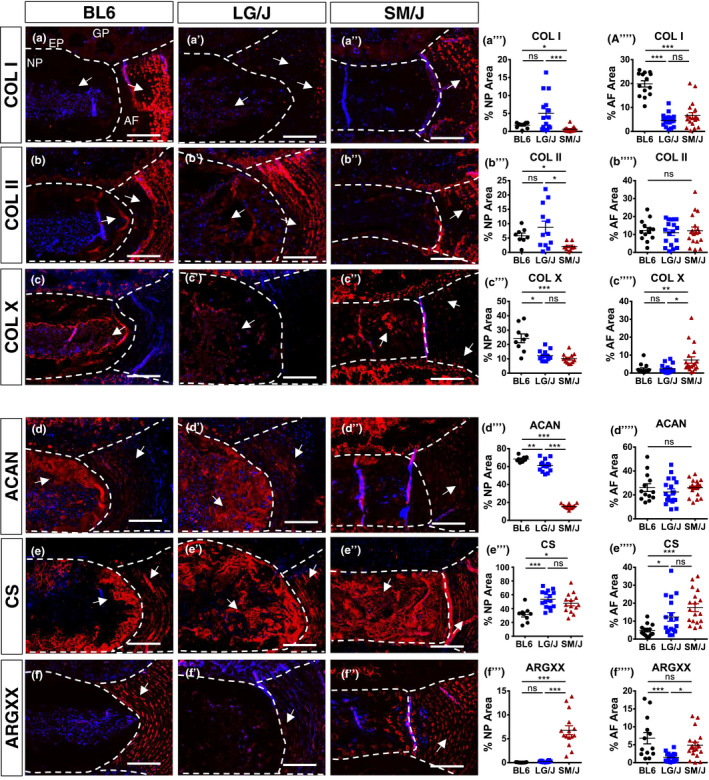
Aged inbred mouse strains evidenced changes in matrix composition. Staining and abundance in NP and AF compartments of important extracellular matrix proteins in 23M BL6, LG/J and SM/J caudal discs (a‐a’’’’) collagen I, (b‐b’’’’) collagen II, (c‐c’’’’) collagen X, (d‐d’’’’) aggrecan, (e‐e’’’’) chondroitin sulphate and (f‐f’’’’) ARGXX neoepitope. ANOVA or Kruskal–Wallis test was used as appropriate; *n* = 6 mice/strain, 2–3 levels per mouse were analysed. Scale bar A‐F’’=50 µm

We assessed the differences in disc matrix composition in 3 strains at 23M. Old SM/J showed the least amount of collagen I in NP whereas both SM/J and LG/J had comparable but lower abundance in the AF (Figure 3a‐a’’’’). Collagen II showed higher levels in both LG/J and BL6 NP than SM/J, without significant strain‐dependent differences in collagen II abundance in the AF (Figure 3b‐b’’’’). Interestingly, collagen X, hypertrophic chondrocyte marker expressed during disc degeneration (Choi et al., 2018), was higher in NP of BL6 compared to SM/J and LG/J (Figure 3c‐c’’’’). However, we also observed more pronounced peri‐cellular staining of collagen X in the SM/J NP, in addition to higher AF levels compared to LG/J and BL6. These results showed that unique collagen signatures underscored disc aging phenotypes in these three strains (Choi et al., 2018).

We examined aggrecan content and functionality parameters. LG/J and BL6 mice showed higher aggrecan abundance compared to SM/J (Figure 3d‐d’’’’). Interestingly, BL6 showed the least chondroitin sulphate staining in both compared to LG/J and SM/J (Figure 3e‐e’’’’). Additionally, SM/J mice showed higher ARGXX neoepitope abundance in the NP compared to both LG/J and BL6 mice, with increased and comparable AF levels to LG/J and BL6, respectively (Figure 3f‐f’’’’). These results suggested that each strain at 23M exhibit a different aggrecan functionality profile.

### 2.4 LG/J, SM/J and BL6 showed divergent transcriptomic profiles

**FIGURE 4:**
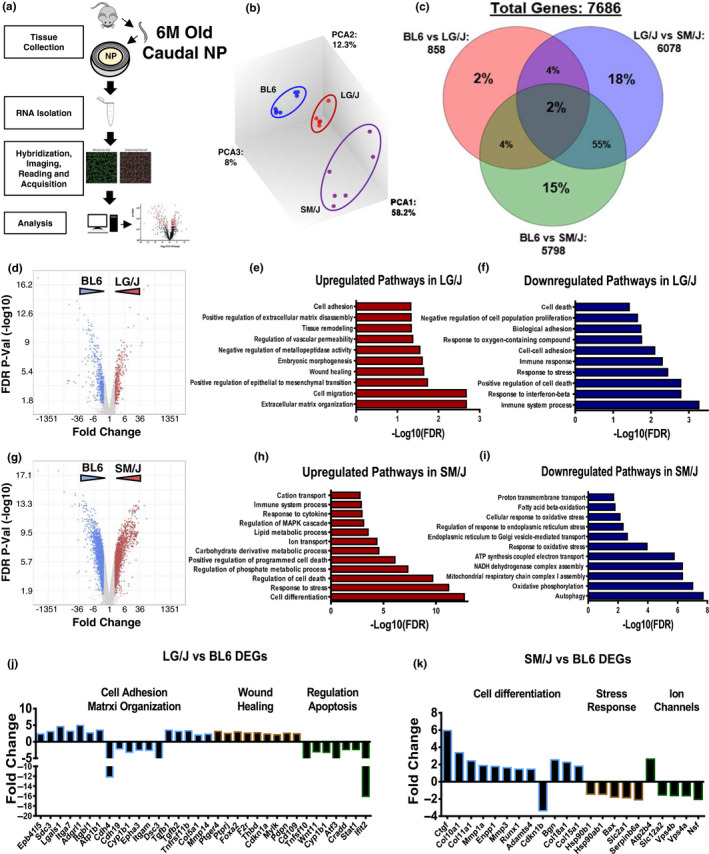
SM/J and LG/J showed divergent transcriptomic profiles at 6M. (a) Schematic summarizing microarray analysis. (b) Transcriptomic profiles of 6M BL6 (*n* = 7), LG/J (*n* = 5) and SM/J (*n* = 6) mice clustered distinctly along principal components. (C) Venn diagram from DEGs, Fold Change ≥ 2, FDR ≤ 0.05, (d) volcano plot, showing up‐ and downregulated DEGs from LG/J versus BL6 comparison, used for GO process enrichment analysis, (e) representative GO processes of upregulated genes in LG/J versus BL6, (f) representative GO processes of downregulated genes in LG/J versus BL6. (g) Volcano plot, showing up‐ and downregulated DEGs from SM/J versus BL6 comparison. (h) Representative GO processes of upregulated genes in SM/J versus BL6. (i) Representative GO processes of downregulated genes in SM/J versus BL6. GO analysis was performed using PANTHER overrepresentation test, GO database annotation, binomial statistical test with FDR ≤ 0.05. Representative genes from select GO processes different between (j) LG/J versus BL6 and (k) SM/J versus BL/6

We performed microarray analysis of NP tissues from 6M LG/J, SM/J and BL6 mice (Figure 4a). The transcriptomic profiles of each strain clustered distinctly along principal components (Figure 4b). Analysis of differentially expressed genes (DEG) using FDR < 0.05 and Fold Change ≥ 2 showed that the highest percentage of DEGs (55%) belonged to SM/J. Since BL6 showed lowest grades of degeneration than both LG/J and SM/J, and LG/J and SM/J had comparable degeneration scores, we performed gene enrichment analysis using PANTHER overrepresentation test (Mi et al., 2019) using BL6 as reference. Volcano plot from LG/J versus BL6 comparison showed distribution of 863 DEGs used for enrichment analysis (Figure 4d). LG/J upregulated DEGs showed enrichment of extracellular matrix organization, wound healing, tissues remodelling and cell adhesion (Figure 4f). Downregulated DEGs in LG/J were enriched for immune system processes, response to stress, cell adhesion as well as cell death (Figure 4f). Comparison of SM/J versus BL6 showed 5,797 DEGs (Figure 4g). SM/J‐upregulated DEGs were enriched for cell differentiation, cell death, ion transport, MAPK cascade and response to cytokines pathways (Figure 4h), whereas SM/J‐downregulated DEGs were enriched for autophagy, oxidative phosphorylation, response to oxidative stress, endoplasmic stress, fatty acid β oxidation and proton transmembrane transport (Figure 4i). To gain further insights, we queried select genes that contributed to these enrichments. In LG/J, we found that adhesion‐related genes Itga7, Itgbl1, Lgals1 and Sdc3 were upregulated along with matrix modulator genes Tgf1, Tgf2, Col5a and Mmp14 (Figure 4j). We also noticed upregulation of hypertrophic chondrocyte markers Ctgf, Col10a1 and Runx1; downregulation of stress factors Hsp90a1, Hsp90b1, Slc2a1 (GLUT1) and ion channels Slc12a2, Vps4b and Vps4a in SM/J mice as previously reported (Choi et al., 2018).

### 2.5 Comparison of aging BL6 and LG/J mice uncovers biological pathways that are independent and dependent on the strain background

**FIGURE 5:**
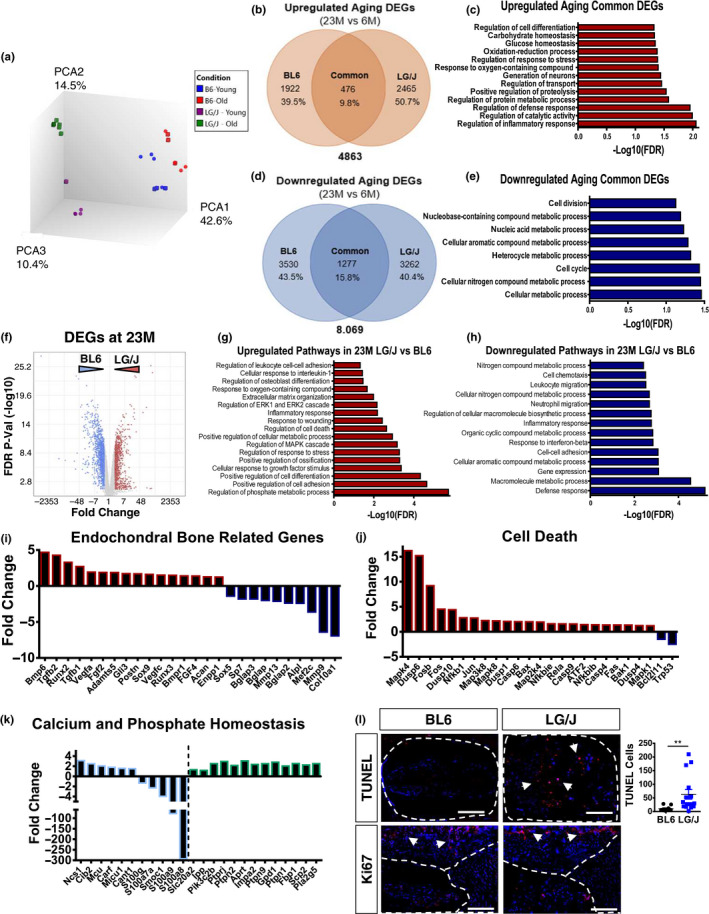
BL6 and LG/J mice comparison uncovers biological pathways that are independent and dependent on the strain background. (a) Transcriptomic profiles of 6M BL6 (*n* = 7), 23M BL6 (*n* = 7), 6M LG/J (*n* = 5) and 23M LG/J (*n* = 5) clustered distinctly along principal components. (b) Venn diagram of upregulated DEGs from BL6 23M versus 6M and LG/J 23M versus 6M, FDR ≤ 0.05. (c) Representative GO processes of the common upregulated genes between BL6 23M versus 6M and LG/J 23M versus 6M. (d) Venn diagram of downregulated DEGs from BL6 23M versus 6M and LG/J 23M versus 6M, FDR ≤ 0.05. (e) Representative GO processes of the common downregulated genes between BL6 23M versus 6M and LG/J 23M versus 6M. (f) Volcano plot of DEGs from LG/J 23M versus BL6 23M, used for GO Process enrichment analysis, Fold Change > 2, FDR ≤ 0.05. (g) Representative GO processes of upregulated genes in LG/J 23M versus BL6 23M. (h) Representative GO processes of downregulated genes in LG/J 23M versus BL6 23M. (i‐k) Representative DEGs from selected GO processes from 23 M LG/J versus 23M BL6. (l) TUNEL (arrows) and Ki67 staining of 23M LG/J and BL6 discs. *t* test or Mann–Whitney test was used as appropriate. *n* = 6 mice/strain were analysed

To explore the mechanisms characterizing disc aging and dystrophic mineralization in LG/J mice, we performed microarray analysis of NP tissues from 23M BL6 and LG/J mice and compared it to 6M data from respective strains. Lack of enough viable cells in SM/J NP precluded their analysis at 23M (Figure S3) (Choi et al., 2018). The transcriptomic profiles of each group clustered distinctly along principal components (Figure 5a). Analysis of DEGs with aging showed a limited overlap of 9.8% in upregulated and 15.8% in downregulated genes between strains. To first explore the common aging‐related DEGs, we perform enrichment analysis of up‐ and downregulated genes that change in both LG/J and BL6 aging (23M versus 6M). The common upregulated DEGs showed an enrichment of inflammatory process, stress and cell differentiation (Figure 5c and Figure S4a‐b) while downregulation of common DEGs related to cell metabolism and cell division was noted (Figure 5e and Figure S4c). To study the crosstalk between the aging phenotype and transcriptomic progression specific to LG/J and BL6 mice, we individually analysed the aging pathways and DEGs in each of the strains. Upregulated genes in old BL6 showed an enrichment of pathways related to blood circulation, ATP and oxidation–reduction processes, calcium ion homeostasis and inflammation response (Figure S5a‐b and d), whereas downregulated BL6 DEGs were enriched in immune response and regulation of gene expression (Figure S5c and e). LG/J‐specific aging DEGs (Figure S6a) were enriched for cell differentiation, response to stress, inflammation, cell death, cell cycle, phosphate metabolism and glucose homeostasis (Figure S6b and d). While downregulated LG/J DEGs enriched into DNA repair, cell cycle and immune response‐related processes. We then explored how LG/J differed from BL6 at 23M, (Figure 5f) and found that upregulated DEGs were enriched for phosphate metabolism, cell differentiation, response to stress, cell death, inflammation response, wound healing, matrix organization and osteoblast differentiation (Figure 5g). Similarly, downregulated DEGs were associated with immune response, cell–cell adhesion, inflammation and leucocyte migration (Figure 5h). Specifically, among enriched pathways, endochondral bone‐related DEGs showed increased levels of BMP6, Tgfb2, Runx2, Fgf2 and Postn, but a decrease in Sp7, Bglap Alpl and Col10a1 (Figure 5i and Figure S7d‐f). Related to cell death, LG/J showed increased expression of Mapk4, Dusp6, Fosb, Fos, Casp6, Casp4 and Rela and decreased Bcl2 (Figure 5j and Figure S7c). We also found increased expression of several key genes related to phosphate/calcium homeostasis and decreased expression of those involved in calcium binding (Figure 5k and Figure S7f). These results suggest that although the strains showed some commonality during aging, strain‐dependent biological processes have a higher contribution to the transcriptomic signature of aged NP cells.

### 2.6 LG/J shows increased cell death without changes in NP marker expression

We performed TUNEL assay to investigate whether there was increased cell death in aged LG/J mice. There were increased number of TUNEL‐positive cells in LG/J mice with a concomitant decrease in survival molecule p21 (Novais et al., 2019), and little change in Ki67 levels. Despite increased cell death, the total number of cells in NP was comparable between LG/J and BL6 (Figure 5l and Figure S7a‐b). Levels of NP markers CA3 and GLUT1 were similar between the two strains but a complete absence of staining was seen in SM/J (Choi, 2018) (Figure S7a).

### 2.7 Inbred strain comparison shows differential effects of aging on vertebral bone parameters

Previous report suggested that LG/J mice have superior bone quality parameters compared to SM/J and BL6 mice (Sabsovich, 2008). To assess the impact of aging on vertebral health, we compared the bone morphology in the caudal spine of 3 strains using μCT (Figure S1a). Although significant changes in the vertebral height were found between the strains, the disc height index (DHI) was comparable at 23M (Figure S1b). There was reduction in trabecular bone parameters in LG/J and SM/J compared to BL6 at 23M, with decrease in BV/TV, trabecular thickness, and number with an increase in trabecular spacing (Figure S1c‐d). Cortical bone analysis showed higher bone volume, thickness, closed porosity and mean polar inertia in LG/J mice compared to both BL6 and SM/J. Additionally, SM/J presented a decrease in cross‐sectional thickness along with an increase in closed porosity compared to BL6 and LG/J mice (Figure S1e‐f). Together, μCT analysis showed that although there were small differences among the strains at 6M, by 23M, the aged SM/J mice evidenced the lowest values for parameters defining vertebral bone composition, whereas LG/J mice showed lower values of trabecular bone than BL6 but thicker and more resistant cortical bone than the two strains.

### 2.8 Degenerated human discs show distinct transcriptomic profiles and enriched biological pathways found in aging mouse strains

**FIGURE 6:**
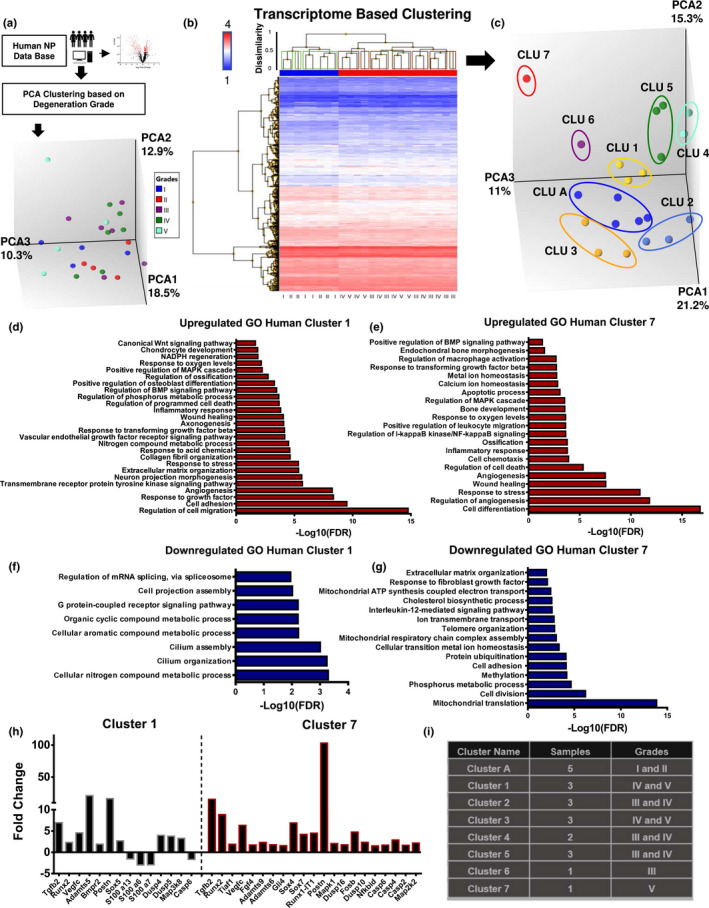
Degenerated human discs show distinct transcriptomic profiles and cluster 1 and 7 present similar enriched pathways with LG/J 23M. (a) Schematic showing PCA clustering of http://www.ncbi.nlm.nih.gov/geo/query/acc.cgi?acc=GSE70362 deposited microarray data based on histological grades (b) Hierarchical clustering of DEGs, *p* ≤ .05, showed 7 degenerative groups—red boxes, and 3 healthy groups—green boxes, with dissimilarly < 0.5 cut‐off Euclidean distance. (c) PCA of clusters obtained in (B). (d) Representative GO processes of upregulated genes between cluster 1 (degenerated) and cluster A (healthy), FDR ≤ 0.1. (e) Representative GO processes of upregulated genes between cluster 7 (degenerated) and cluster A (healthy), FDR ≤ 0.1 (f) Representative GO processes of downregulated genes between cluster 1 and cluster A FDR ≤ 0.1. (g) Representative GO processes of downregulated genes between cluster 7 and cluster A, FDR ≤ 0.1 (h) Representative DEGs in comparisons from cluster 1 versus cluster A and cluster 7 versus cluster A (i) Representative table of the cluster details used for enrichment analysis

To understand the relationship between mouse models and human pathology, we re‐analysed microarray data set of healthy and degenerated human NP tissues from a previous study by Kazezian et al. (2015) (http://www.ncbi.nlm.nih.gov/geo/query/acc.cgi?acc=GSE70362). Interestingly, the transcriptomic profiles of healthy versus degenerated discs did not cluster distinctly according to standard histological grades of degeneration (Figure 6a). Therefore, using a hierarchical clustering of transcriptomes of all the available healthy (grade 1 and 2—in blue) and degenerated (grades 3, 4 and 5—in red) NP samples, with a cut‐off of dissimilarity < 0.5 (Euclidean distance), we categorized the samples into 7 degenerative groups—red boxes, and 3 healthy groups—green boxes (Figure 6b). From these groups, we compared the cluster A (healthy cluster with most samples—5) to the remaining 7 degenerative clusters. This new analysis showed that each cluster organized together along the principal components (Figure 6c). To further explore the differences and similarities of gene expression from these cluster comparisons, we performed enrichment biological processes analysis of both upregulated and downregulated DEGs. Due to the divergent transcriptome signature of each degenerative cluster, we found that while some enriched pathways were similar, each cluster also presented unique enriched pathways (Figure 6d‐G and Figure S8). Moreover, not all the clusters showed enrichment of up‐ and downregulated DEGs in biological pathways. Interestingly, similar to LG/J mice, clusters 1 and 7 presented upregulated DEGs enriched for response to stress, wound healing, cell death, endochondral bone and inflammation pathways (Figure 6d‐e). While downregulated DEGs from cluster 1 were enriched for nitrogen metabolic process, cilium organization and assembly and G protein‐coupled receptor signalling pathway, cluster 7 shown altered mitochondria homeostasis, cell division, phosphorous metabolism and extracellular matrix organization (Figure 6f.g). We found that genes that changed in aged LG/J (vs. BL6) such as TGFb1, TGFb2, Runx2, Adamts5, Postn, Dusp4, Casp4 and Casp6 were present in both clusters 1 and 7 (Figure 6h). Lastly, we performed enrichment analysis on DEGs from comparisons of remaining degenerative clusters with cluster A (Figure 6i). By analysing the upregulated DEGs, we found that cell death was found in 4 different clusters; cell differentiation, angiogenesis and response to stress in 3; inflammation and response to oxygen in 2 clusters; regulation of macrophages and phosphorus in 1(Figure 6d‐e and Figure S8), whereas downregulated DEGs were enriched for nitrogen metabolism in 4 clusters; mitochondria homeostasis/respiration, cell division and ion/intracellular transport in 3; cell adhesion, DNA repair/telomere organization, cilium organization and NADH dehydrogenases regulation in 2 clusters; extracellular matrix and phosphorous metabolism in 1 (Figure 6f‐g and Figure S8). These results suggested that disc degeneration is not a uniform molecular process; but, similar to wide spectrum of degenerative morphological phenotypes (Cheung et al., 2009), it is underscored by multiple molecular signatures.

## 3 DISCUSSION

While disc degeneration is complex and thought to have contributions from a multiple factors, aging is considered one of the major risk factors (Novais et al., 2019; Ohnishi et al., 2018). Human disc degeneration has diverse phenotypes ranging from loss of disc height, herniations and disc calcification (Cheung et al., 2009); however, the contribution of genetic background to these aging phenotypes is not well understood. Commonly used animal models with disc injury or genetic modifications have recapitulated some of these phenotypes, but do not provide insights why aging has such a wide spectrum of disc degeneration outcomes (Choi et al., 2018; Novais et al., 2019). In this study, we show that the disc aging phenotype is dependent on the strain background in mice. While BL6 and SM/J mice present with a mild to fibrotic degeneration, respectively, LG/J mice, recapitulate several aspects of human age‐dependent disc calcification and evidence aging transcriptomic changes that are both dependent and independent of the strain background.

BL6 mice are considered gold‐standard model for aging studies and are widely used to characterize the aging of the spine (Novais et al., 2019; Ohnishi et al., 2018). It is well known that human disc aging and degeneration comprises a wide spectrum of phenotypes, many of which are not recapitulated in the BL6 model, that is disc calcification (Chanchairujira et al., 2007; Cheung et al., 2009). Interestingly, μCT analysis of LG/J mice showed ~80% prevalence of disc calcification in the caudal spine of old mice. Histological studies showed that this calcification was widespread and affected both the NP and AF. Additionally, presence of high concentration of free calcium not associated with a mineralization nodules and matrix proteins suggested its dystrophic nature. While studies of single gene knockouts affecting PPi metabolism showed ectopic mineralization of the AF and adjacent ligaments (Warraich et al., 2013; Zhang et al., 2016) LG/J mice recapitulated age‐dependent disc calcification that affected both the NP and AF similar to elderly humans (Chanchairujira et al., 2007). Studies have also shown a causal relationship between systemic activity of TNAP that degrades PPi into Pi and ectopic calcification of soft tissues (Ziegler et al., 2017). Interestingly, blood analysis showed that several mineral metabolism markers including free Ca^2+^ and phosphorous were not affected by the strain background. While TNAP levels increased with aging in LG/J mice so did they in SM/J mice which also showed decreased levels of fetuin, a known inhibitor of ectopic calcification (Schäfer et al., 2003). Despite these differences in TNAP and fetuin, SM/J mice did not evidence disc mineralization suggesting that systemic mineral metabolism had little effect on the intradiscal mineralization. Importantly, a previous study showed that LG/J mice despite having higher articular cartilage healing potential, evidenced increased susceptibility to synovial and meniscus calcifications following knee trauma (Rai, Schmidt, Hashimoto, Cheverud, & Sandell, 2015). Our results are in line with these findings that stressors like trauma and aging may promote mineralization in LG/J mice. Moreover, there was a higher incidence of calcification at Ca6‐7, Ca7‐8 and Ca8‐9, whether increased mechanical/loading stress at these levels could be a contributing factor remains to be seen. Additionally, our results clearly showed that unlike caudal discs, lumbar discs of LG/J mice do not show calcification underscoring the importance of anatomic location, and consequently local mechanical environment, in predisposition to a degenerative phenotype (Teraguchi et al., 2014).

BL6, LG/J and SM/J discs showed different tissue and cellular organization with aging. Interestingly, BL6 caudal discs showed little increase in degeneration grades with aging as reported for lumbar and cervical spine (Ohnishi et al., 2018). This is not surprising as in humans, prevalence of disc degeneration is higher at lumbar L5/S1 and cervical C5/C6 than the other levels, suggesting that factors such as the local mechanical environment could play an important role in increasing the susceptibility to degeneration (Teraguchi et al., 2014). While SM/J mice presented higher grades of degeneration at 6M (Choi et al., 2018), they showed progressive changes with an increase in NP fibrosis. This was not unexpected since at 17 week, SM/J mice were shown to have fewer NP cells, and those remaining exhibited a hypertrophic chondrocyte‐like phenotype responsible for secretion of collagenous fibrotic matrix (Choi et al., 2018). While most hypertrophic cells in NP did not survive at 23M, early changes in cell fate supports an overall decrease in aggrecan content (Choi et al., 2018). We also observed an accumulation of ARGXX neoepitope in the AF of old SM/J suggesting increased aggrecanase activity Sivan, Wachtel, & Roughley, 2014), whereas at 6M, LG/J mice showed only a small increase in disc degeneration compared to BL6; however, the divergence was more pronounced with aging. Likewise, despite comparable grades of degeneration to SM/J at 23M, the LG/J phenotype was completely distinct in terms of matrix dynamics and mineralization. Previous studies have established a relationship between increased fibrosis, elevated matrix degradation and decreased aggrecan content with increased intervertebral disc stiffness and loss of mechanical properties (Choi et al., 2018) underscoring plausible functional differences between these strains. These results suggested a strong reliance of age‐dependent disease progression and phenotypic presentation on the genetic background.

Animal studies have largely overlooked the contribution of genetic background on disc aging phenotypes (Gorth, Shapiro, & Risbud, 2019; Novais et al., 2019; Tessier, Tran, Ottone, & Novais, 2019). This is important since previous studies with twins’ cohorts have reported a strong correlation between familiar aggregation and disc degeneration (Battié, 2009; Munir et al., 2018). Along with the higher grades of disc degeneration seen in SM/J at 6M, they differed the most in terms of gene expression compared to BL6 and LG/J. This suggested that severity and rate of progression of morphological changes were reflected in magnitude of changes at transcriptomic levels. Interestingly the genes and pathways that altered in SM/J such as cell differentiation, cell death, ion and proton transport and metabolic processes supported previous observations in this model (Choi et al., 2018). Surprisingly, although we found common DEGs between BL6 and LG/J with aging, most of them were divergent between the strains. Alterations in genes that are associated with inflammatory processes, response to stress, cell differentiation, cell metabolism and cell division were shared. Recent aging studies supports the importance of inflammation and metalloproteases secretion (Novais et al., 2019; Tessier et al., 2019), cell differentiation (Mohanty, Pinelli, Pricop, Albert, & Dahia, 2019) and cell cycle (Novais et al., 2019; Patil et al., 2019) in disc health. Moreover, comparison of altered pathways in LG/J aging with changes seen between old LG/J versus BL6, suggested that 23M LG/J mice evidence similar changes in cell differentiation/ossification, inflammation, cell death, phosphate metabolism along with altered DNA repair and immune response‐related processes. While enrichment analysis could not establish causality, they showed a strong association with observed phenotype providing plausible insights into mechanistic underpinning of the intradiscal calcification. Supporting the contribution of cell death and elevated free local calcium and phosphate to soft tissue mineralization, previous studies have shown linked increased tissue cell death to dystrophic calcification (Priante et al., 2019). While resident cells in old LG/J mice expressed NP markers, GLUT‐1 and CA3, they also increased expression of endochondral genes BMP6, Tgfb2, Runx2, Fgf2 and Postn with a concomitant decrease in terminal osteoblastic differentiation markers Sp7, Bglap and Alpl. Despite the absence of lineage tracing, results suggested the possibility that the resident‐NP cells in old LG/J were not true osteoblasts. This idea is supported by a study that showed limited osteoblast‐like characteristics by smooth muscle cells in the context of ectopic arterial calcification (Patel et al., 2019). Concerning increase in macrophage markers CD14, CD68 and CD163, studies have shown a strong link between macrophage activation with dystrophic calcification (Chen et al., 2016) and bone healing (Vi et al., 2015). Noteworthy, our recent results showed that NP cells possess immune cell‐like properties and process extracellular antigens (Gorth, Shapiro, et al., 2019).

Histological analysis of human tissues have described various morphological features: disc fibrosis with acellularity, annular clefts, neovascularization, sclerosis of the subchondral bone; ectopic calcification of the endplate and/or the disc itself; herniation with increased senescent cells (Roberts, Evans, Trivedi, & Menage, 2006). However, the contribution of these abnormalities in the etiopathogenesis of disc disorders as well as their actual cause—whether genetic predisposition, tissues response or altered mechanical environment—is not clear. To elucidate the genetic signatures of these phenotypes, we analysed the transcriptomic profiles of nondegenerated and degenerated human discs. Our analysis resulted in 7 distinct clusters of disc degeneration with unique molecular pathways. We found that several altered pathways in 6‐month‐old SM/J mice were represented in several human degenerated clusters: upregulation of cell death in 4 different clusters; cell differentiation, and response to stress in 3; and changes in metabolism and ion/intracellular transport in 3 clusters. In addition, inflammation and immune processes altered during BL6 aging were seen in some human clusters. Importantly, disc calcification is shown to be present in about ~6% of degenerated human discs (Chanchairujira et al., 2007). Interestingly, similar to enriched pathways in old LG/J mice, we found two of the human clusters showed enrichment of genes in pathways concerning response to stress, wound healing, cell death, endochondral bone, inflammation, cell division, phosphorous metabolism and extracellular matrix organization. These similarities support the hypothesis that common molecular pathways underscore disc degeneration in mice and humans. In conclusion, by analysing the aging NP signature in inbred mouse strains, as well as uncovering distinct transcriptomic profiles of human disc degeneration, our studies shed light on the wide spectrum of disorders presented in degenerative disc disease. Importantly, this study clearly demonstrated how genetic background can promote specific morphological phenotypes in the disc and highlights the utility of LG/J mice as a model to study intervertebral disc calcification.

## 4 METHODS

### 4.1 Mice

All animal experiments were performed under IACUC protocols approved by the Thomas Jefferson University. 6M and 22‐23M C57BL/6 mice from NIA aged Rodent colony, LG/J (Stock # 000675, Jackson Labs) and SM/J (Stock # 000687, Jackson Labs) mice at 6M and 23M were analysed. While LG/J and SM/J life estimates do not vary considerably (Staats, 1976), no systematic study has provided comparative estimates on cellular senescence activity between these three strains.

### 4.2 Histological Analysis

Dissected spines were fixed in 4% PFA in PBS for 48 hr, decalcified in 20% EDTA and embedded in paraffin. Spines used for calcified sections were fixed for 2h, treated with 30% sucrose, OCT embedded and snap‐frozen. 7 µm mid‐coronal sections were cut from 6 caudal levels (Ca3‐9) of each mouse and stained with Safranin‐O/Fast Green/haematoxylin for assessing histology or Picro‐Sirius Red, to visualize the collagen content. Alizarin Red staining was used to detect calcium. Staining was visualized using a Axio Imager 2 microscope (Carl Zeiss) using 5×/0.15 N‐Achroplan or 20×/0.5 EC Plan‐Neofluar objectives (Carl Zeiss) or a polarizing microscope (Eclipse LV100 POL, Nikon) using 10×/0.25 Pol/WD 7.0 objective, Digital Sight DS‐Fi2 camera, and NIS Elements Viewer software. To evaluate degeneration, mid‐coronal sections from >4 caudal discs per mouse were scored using a modified Thompson Grading scale by 4 blinded observers (Choi et al., 2018).

### 4.3 Immunohistology and cell number measurements

De‐paraffinized sections following antigen retrieval were blocked in 5% normal serum in PBS‐T and incubated with antibodies against collagen I (1:100, Abcam ab34710), collagen II (1:400, Fitzgerald 70R‐CR008), collagen X (1:500, Abcam ab58632), aggrecan (1:50, Millipore AB1031); chondroitin sulphate (1:300, Abcam ab11570); CA3 (1:150, SantaCruz), Ki67 (1:100, Abcam ab15580) and p21 (1:200, Novus NB100‐1941). For GLUT‐1 (1:200, Abcam, ab40084) and ARGxx (1:200, Abcam, ab3773) staining, MOM kit (Vector laboratories, BMK‐2202) was used for blocking and primary antibody incubation. Tissue sections were washed and incubated with Alexa Fluor‐594‐conjugated secondary antibody (Jackson ImmunoResearch Lab, Inc., 1:700). The sections were mounted with ProLong^®^ Gold Antifade Mountant with DAPI (Fisher Scientific, P36934) and visualized with Axio Imager 2 microscope using 5×/0.15 N‐Achroplan or 20×/0.5 EC Plan‐Neofluar objectives (Carl Zeiss). Staining area and cell number quantification was performed using the ImageJ software. Images were thresholder to subtract the background, transformed into binary, and then staining area and cell number were calculated using the analyse particles function.

### 4.4 TUNEL assay

TUNEL staining was performed using “In situ cell death detection” Kit (Roche Diagnostic). Briefly, sections were de‐paraffinized and permeabilized using Proteinase K (20 μg/mL) for 15 min and TUNEL assay was carried out per manufacturer's protocol. Sections were washed and mounted with ProLong^®^ Gold Antifade Mountant with DAPI and visualized with Axio Imager 2 microscope.

### 4.5 Tissue RNA isolation and real‐time RT‐PCR analysis

NP tissue was dissected from caudal discs (Ca1‐Ca15) of 6M SM/J (*n* = 6:4 females, 2 males), 6M and 23M BL6 mice (*n* = 7:4 females, 3 males), LG/J (23M *n* = 5:4 females, 1 males; 23M *n* = 6:6 males) and pooled tissue from single animal served as an individual sample. Samples were homogenized and total RNA was extracted using RNeasy^®^ Mini kit (Qiagen). The purified, DNA‐free RNA was converted to cDNA using EcoDry™ Premix (Clontech). Template cDNA and gene‐specific primers (IDT, IN) were added to Power SYBR Green master mix, and expression was quantified using the Step One Plus Real‐time PCR System (Applied Biosystems). HPRT was used to normalize gene expression. Data are represented as mean ± *SEM* *n* ≥ 4 mice/group.

### 4.6 Microarray analysis and enriched pathways

Total RNA with RIN 5–9 was used for the analysis. Fragmented biotin‐labelled cDNA was synthesized using the GeneChip WT Plus kit according to ABI protocol (Thermo Fisher). Gene chips (Mouse Clariom S) were hybridized with biotin‐labelled cDNA. Arrays were washed and stained with GeneChip hybridization wash and stain kit and scanned on an Affymetrix Gene Chip Scanner 3000 7G, using Command Console Software. Quality control of the experiment was performed by Expression Console Software v 1.4.1. Chp files were generated by sst‐rma normalization from Affymetrix CEL file using Expression Console Software. Only protein‐coding genes were included in the analyses. Detection above background higher than 50% was used for Significance Analysis of Microarrays (SAM) and the false discovery rate (FDR) was set at 5%. Biological process enrichment analysis was performed using PANTHER overrepresentation test, GO database annotation, binomial statistical test with FDR ≤ 0.05. Analyses and visualizations were done in Affymetrix Transcriptome array console 4.0 software. Array data are deposited in the GEO database (http://www.ncbi.nlm.nih.gov/geo/query/acc.cgi?acc=GSE134955).

### 4.7 Human microarray analysis

All sample details were reported by Kazezian et al. (2015) and available in GEO database, http://www.ncbi.nlm.nih.gov/geo/query/acc.cgi?acc=GSE70362 (Kazezian et al., 2015). Degenerative and nondegenerative clusters were obtained by hierarchical clustering of DEGs with a Euclidean distance < 0.5 cut‐off. DEGs used for overrepresented biological processes enrichment analysis using PANTHER overrepresentation test, GO database annotation, binomial statistical test with FDR ≤ 0.05 of up‐ and downregulated DEGs between each degenerated cluster and cluster A (healthy).

### 4.8 Micro‐CT analysis

Micro‐CT (μCT) scanning (Bruker SkyScan 1275) was performed on fixed spines using parameters: 50 kV (voltage), 200 μA (current) at 30 μm resolution. The 3D data sets were assessed for bone volume fraction (BV/TV), trabecular thickness (Tb. Th), trabecular number (Tb. N) and trabecular separation (Tb. Sp). For cortical bone analysis, 2D assessments computed cortical bone volume (BV), cross‐sectional thickness (Cs. Th), polar moment of inertia (MMI) and closed porosity per cent (Po(cl)). Disc height and vertebral length were measured at three different points equidistant from the centre of the bone on the sagittal plane. Disc height index (DHI) was calculated (Choi et al., 2018).

### 4.9 FTIR imaging spectroscopy

IR spectral imaging data in the mid‐IR region, 4,000–800/cm at 8/cm spectral resolution and 25 μm spatial resolution was acquired from 10‐μm‐thick calcified caudal disc sections from 6M and 23M LG/J mice (*n* = 3 discs/group) using a Spectrum Spotlight 400 FT‐IR Imaging system (Perkin Elmer, CT) (Choi et al., 2018; Mohanty et al., 2019). Absorbance for proteins in the amide I region, 1,665/cm; phosphate vibration region, 960 cm^−1^ and the carbonate at 870/cm were recorded (Berzina‐Cimdina & Borodajenko, 2012). The preprocessed spectra were used for K‐means cluster analysis to define anatomical regions and tissue types within the tissue section spectral images (Choi et al., 2018), which represent each analysed peak and ratio. clustering images were obtained using Spectrum Image Software.

### 4.10 Blood analysis

Blood was collected by heart puncture following sacrifice and centrifuged at 1,500 rcf, at 4 degrees, for 15 min to obtain plasma and send to measure analyte levels (IDEXX BioAnalytics). Fetuin‐A was quantified using the mouse Fetuin‐A/AHSG DuoSet ELISA (R&D Systems) according to the manufacturer's instructions.

### 4.11 Statistical analysis

Analysis was performed using Prism7 (GraphPad, La Jolla). Data are represented as mean ± *SEM*. Data distribution was checked with Shapiro–Wilk normality test and the differences between two groups were analysed by *t* test or Mann–Whitney as appropriate. The differences between 3 groups were analysed by ANOVA or Kruskal–Wallis for nonnormally distributed data followed by Dunn's multiple comparison test. Chi‐squared test was used to analyse differences between distribution of percentages. *p* ≤ .05 was considered a statistically significant difference.

## CONFLICT OF INTEREST

Nothing to disclosure.

## AUTHOR CONTRIBUTIONS

EJN, MVR and IMS designed the study. EJN, MVR, VAT, JM, KS, AS, ND, SA, FS and KVW collected and analysed the data. EJN, MVR, IMS, ND, FS and KVW prepared the manuscript.

## Supporting information

FigureS1–S8

Supplementary Material

## Data Availability

Microarray data set that supports the findings of this study are openly available in GEO database, accession number http://www.ncbi.nlm.nih.gov/geo/query/acc.cgi?acc=GSE134955.
