# Supplementary material for: Comparison of inbred mouse strains shows diverse phenotypic outcomes of intervertebral disc aging
Source: Aging Cell. 2020 Apr 22;19(5):e13148. doi: 10.1111/acel.13148 (PMC7253061; doi:10.1111/acel.13148)

Supplementary Figure 1

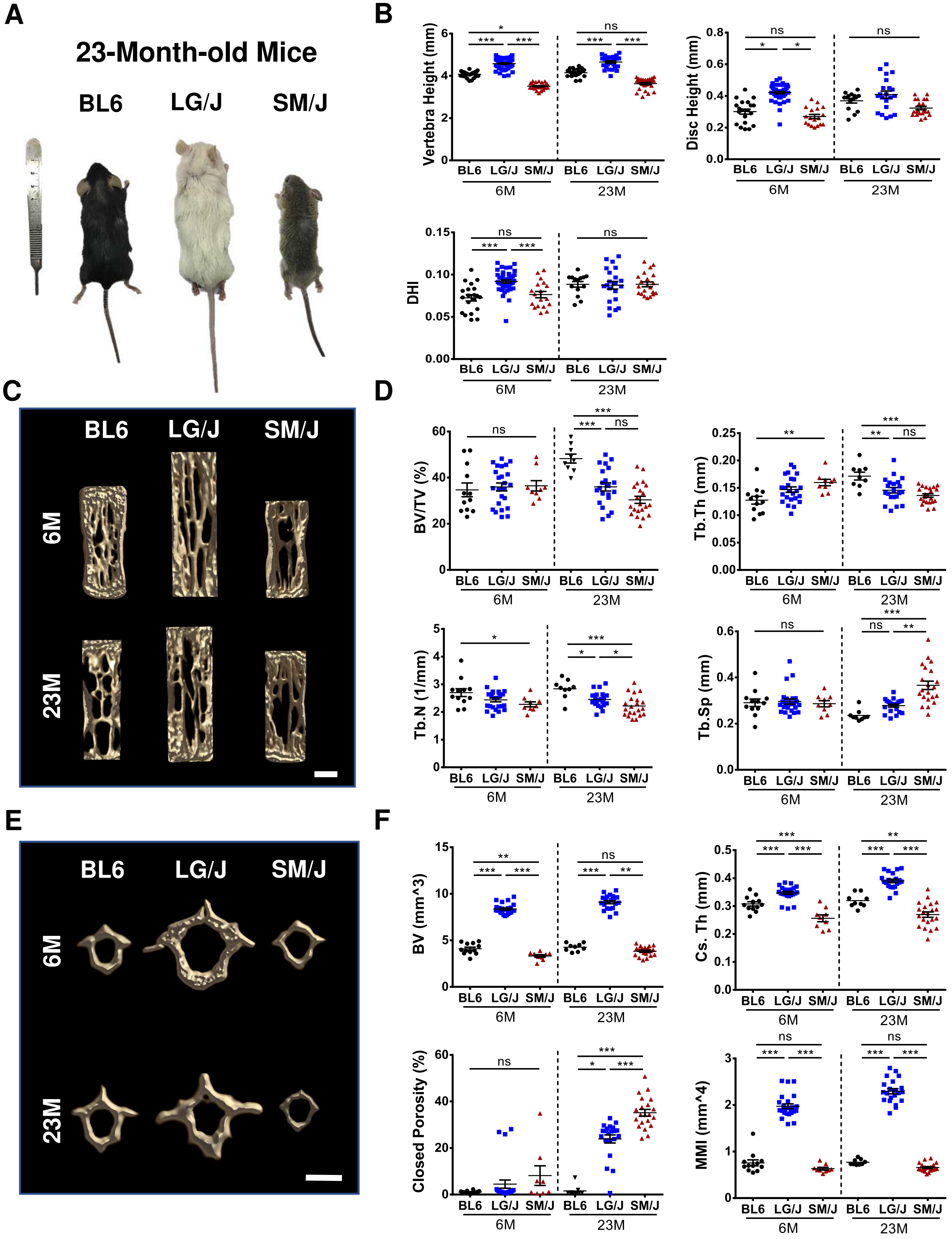

Supplementary Figure 2

A

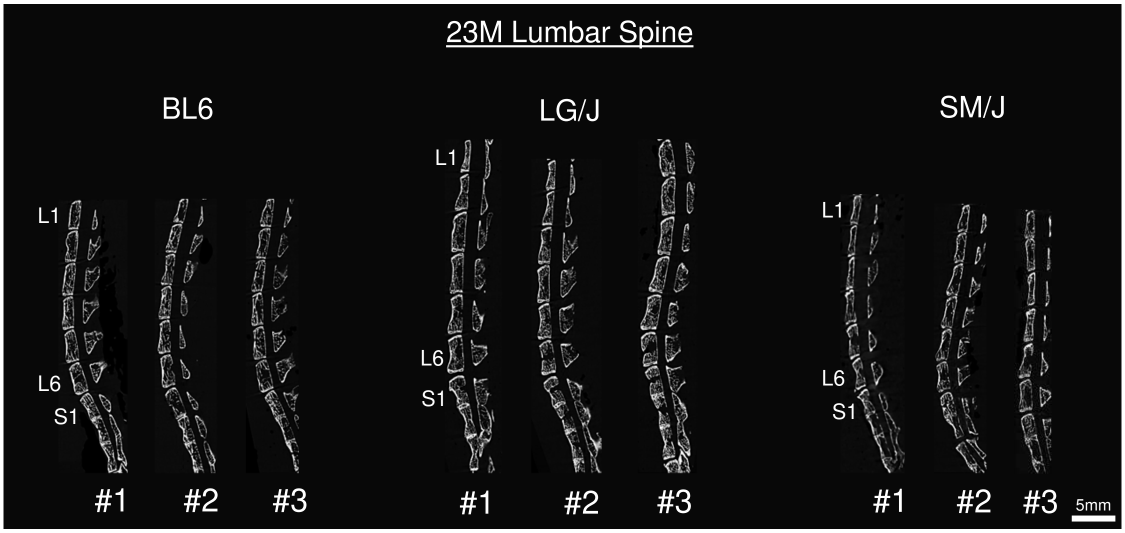

B

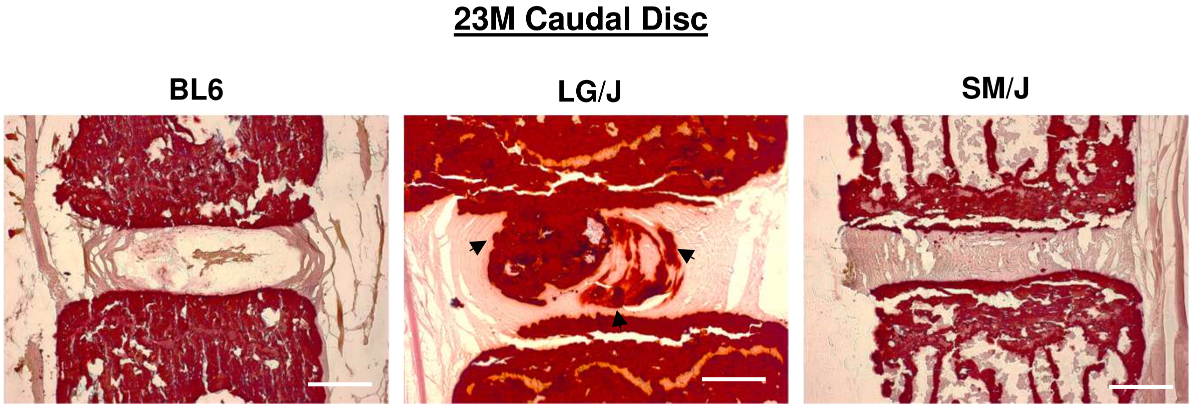

Supplementary Figure 3

A

Modified Thompson Grading

NP Grading:

1. Aggrecan-rich bulging tissue
2. Peripheral fibrous tissue
3. Consolidated fibrous tissue
4. Horizontal clefts parallel to endplate
5. Clefts through NP and AF (herniation)

AF Grading:

1. Discrete fibrous lamellae
2. Mucinous material between lamellae
3. Extensive mucinous material and/or loss of NP-AF demarcation
4. Focal clefts
5. Clefts through NP and AF (herniation)

B

23 MONTH HISTOLOGY

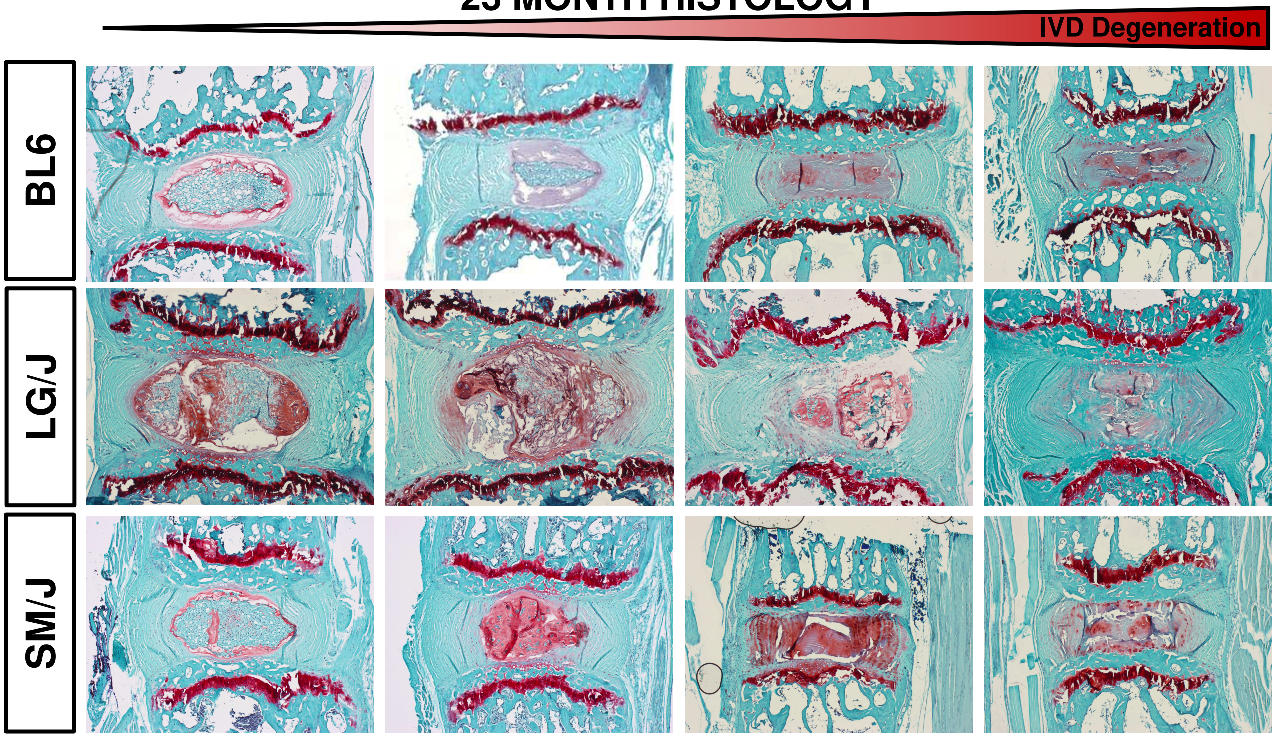

C

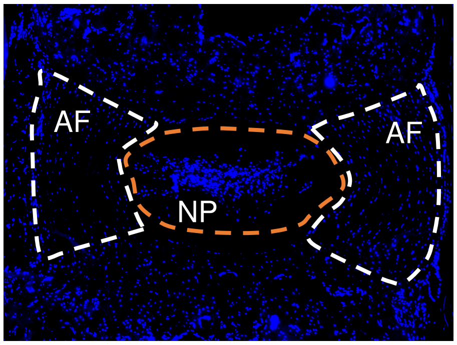

Supplementary Figure 4

A

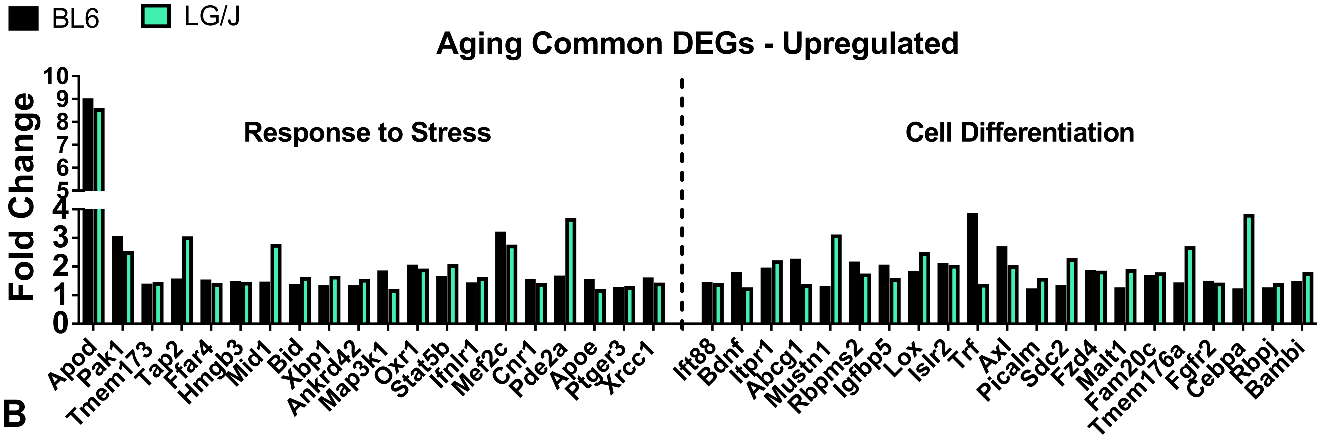

B

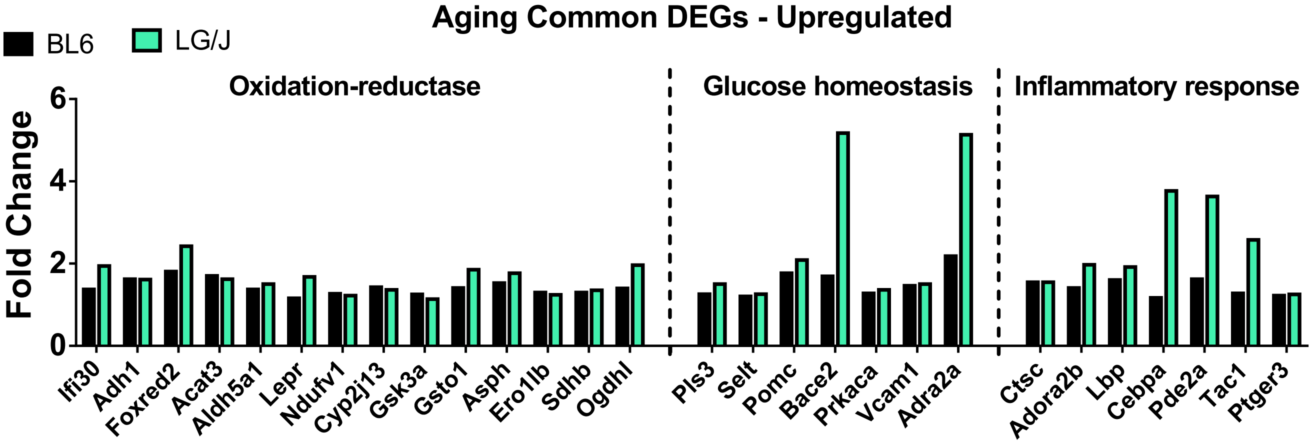

C

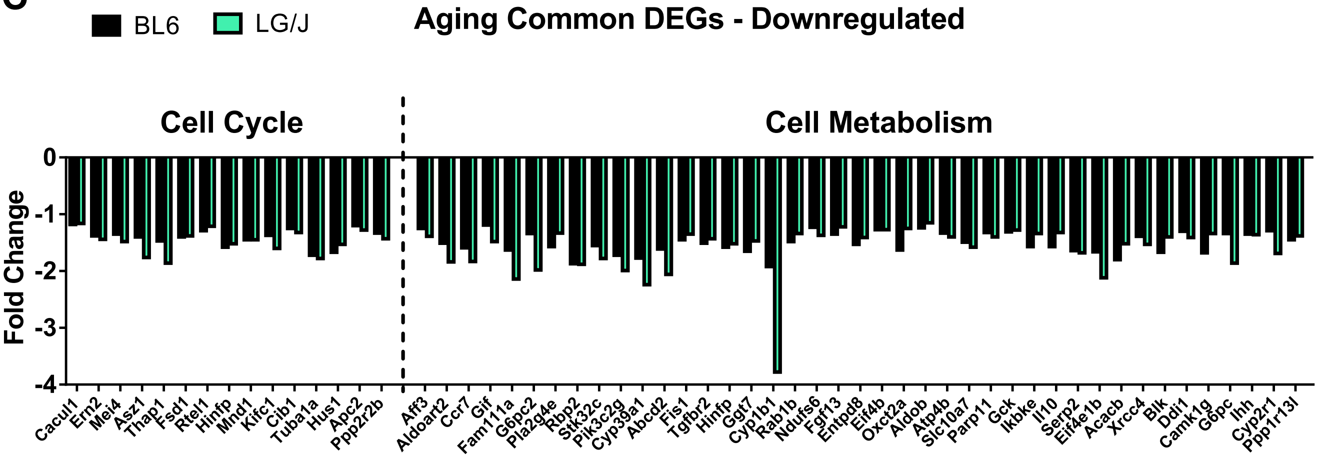

Supplementary Figure 5

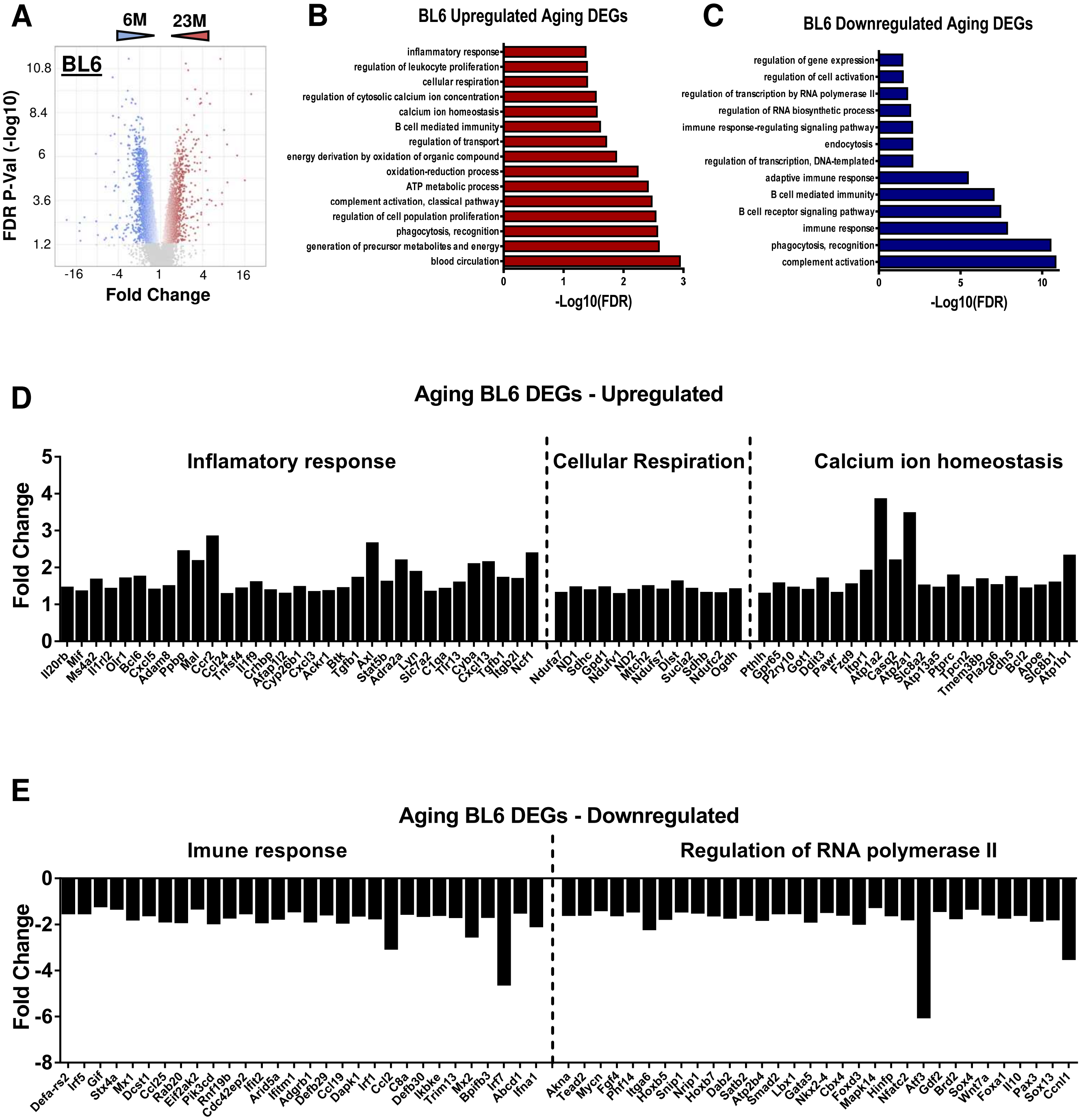

Supplementary Figure 6

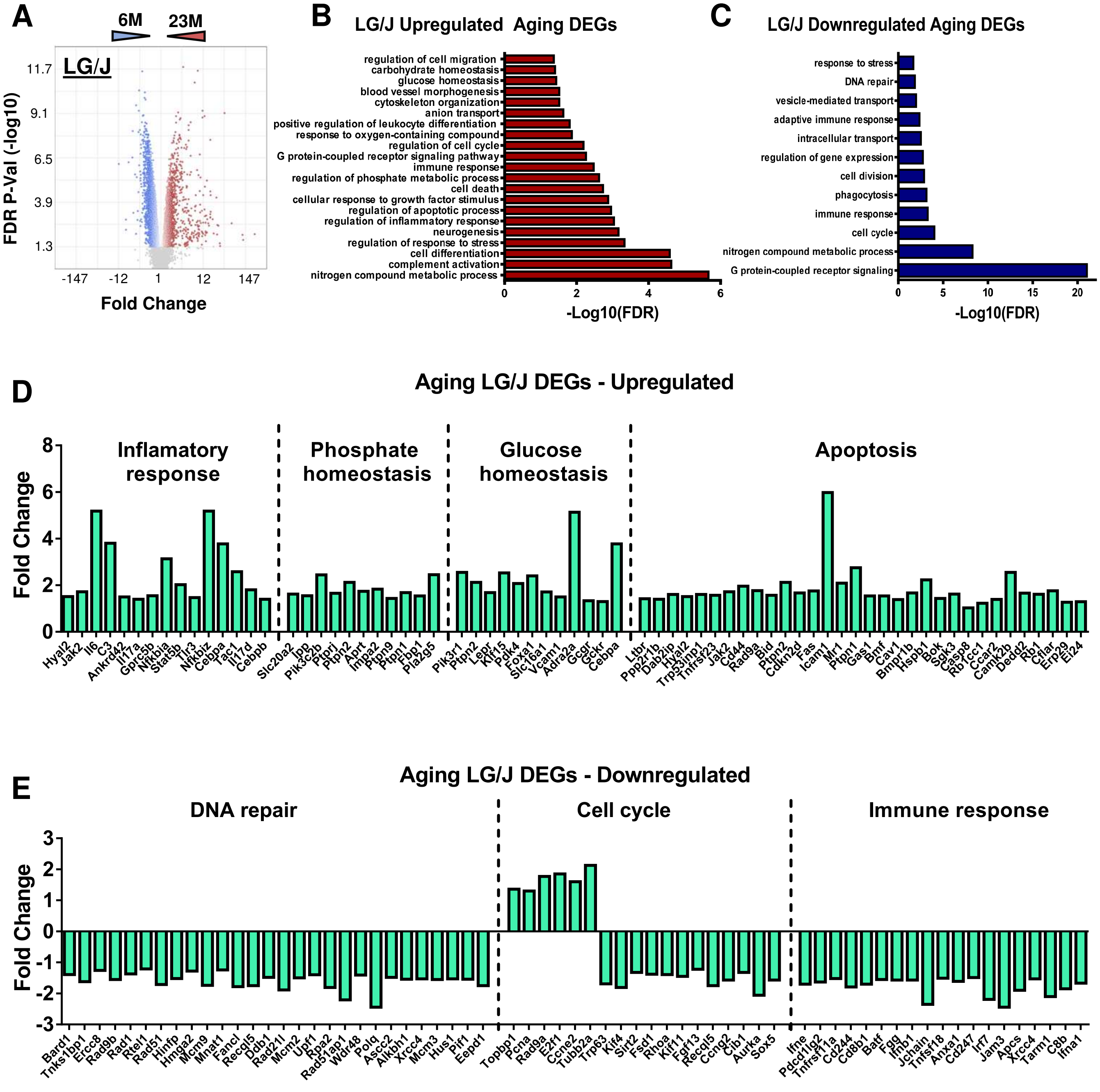

Supplementary Figure 7

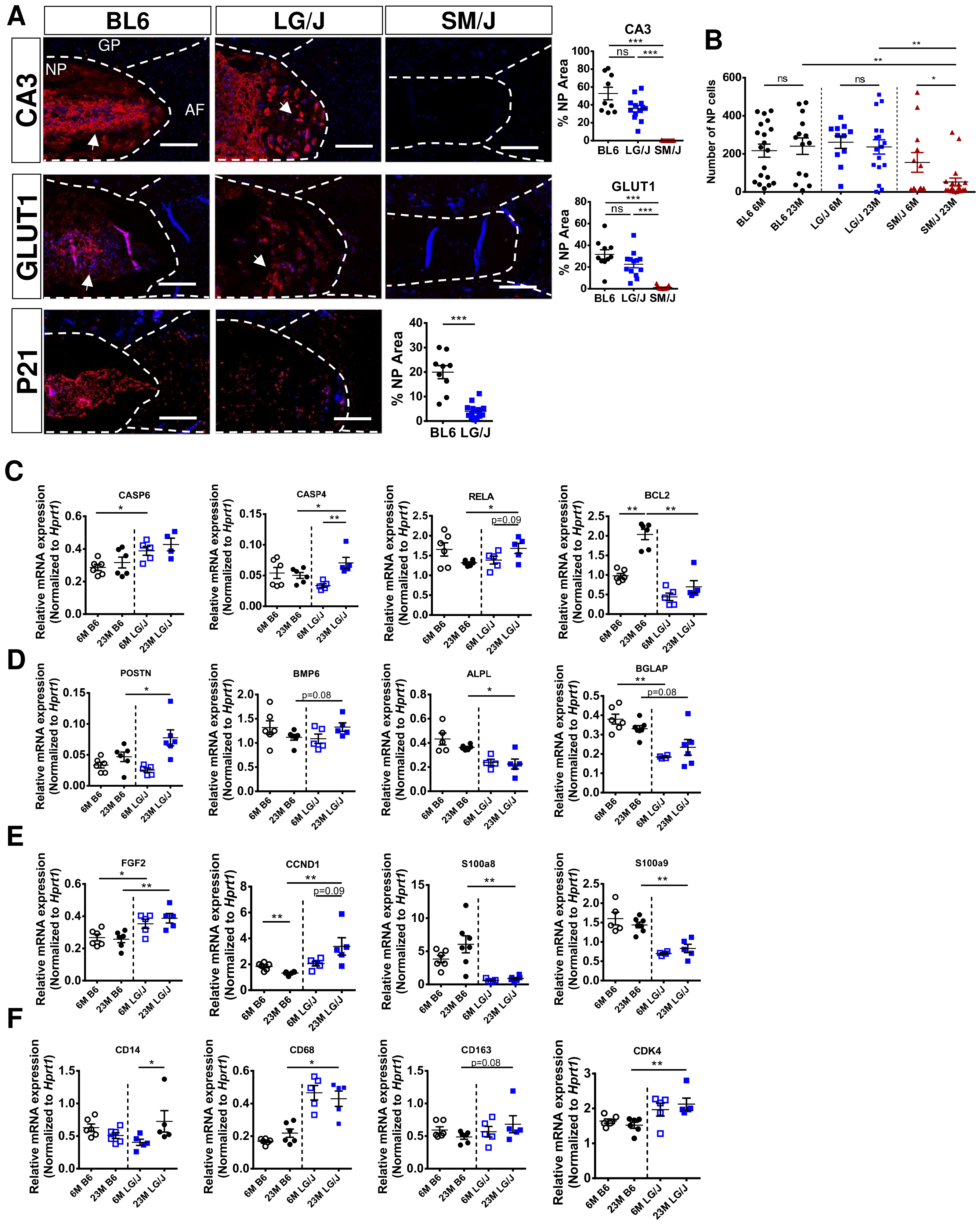

Supplementary Figure 8

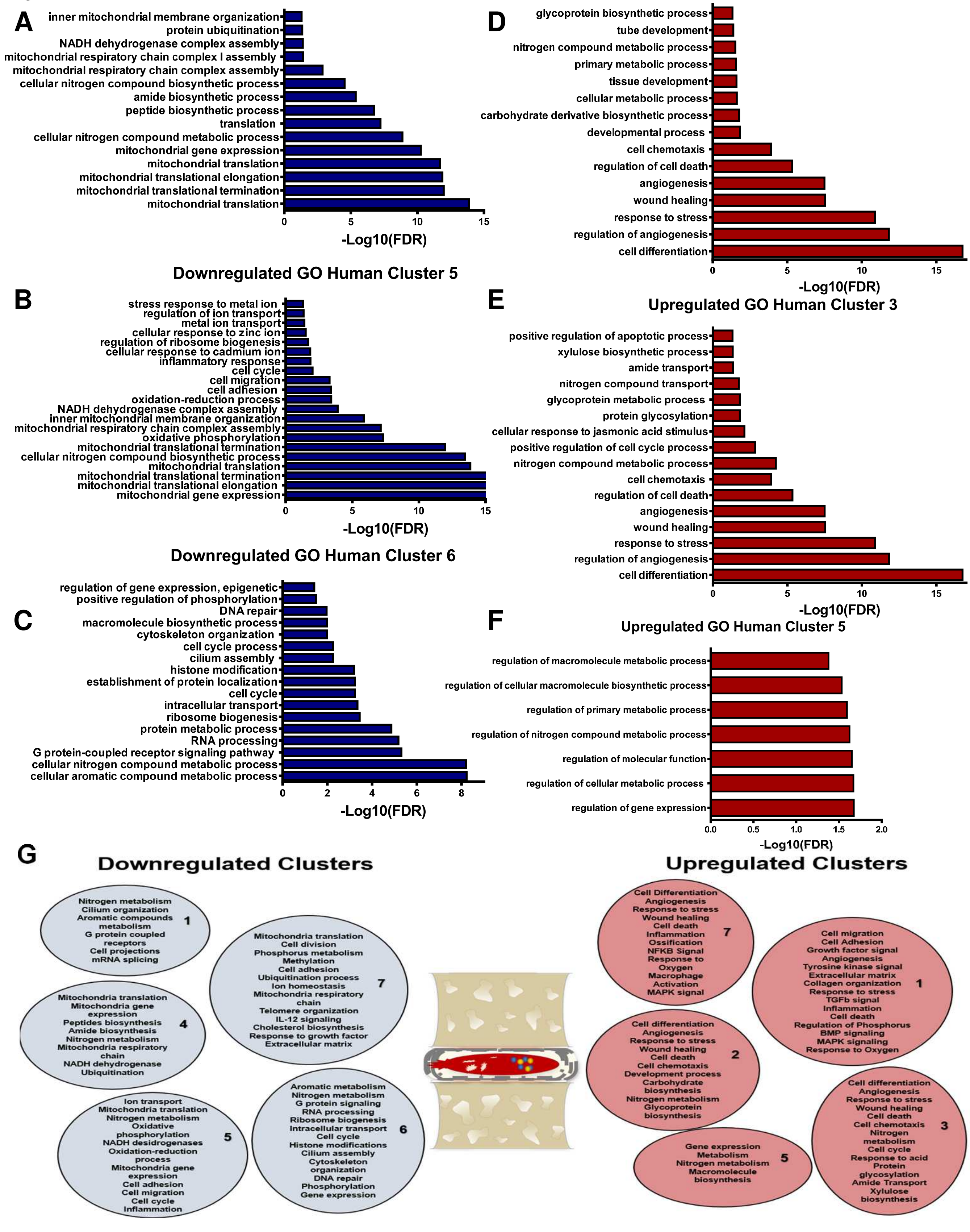

Supplement: Supplementary file 1 — FigureS1–S8 [file ACEL-19-e13148-s001.pdf]
