# Supplementary material for: Comparison of inbred mouse strains shows diverse phenotypic outcomes of intervertebral disc aging
Source: Aging Cell. 2020 Apr 22;19(5):e13148. doi: 10.1111/acel.13148 (PMC7253061; doi:10.1111/acel.13148)
Supplement: Supplementary file 2 — Supplementary Material [file ACEL-19-e13148-s002.pdf]

**Supplemental Figure 1: Inbred strain comparison shows differential effects of aging on vertebral bone. (A)** 23-month-old LG/J have bigger body size than C57BL/6 and SM/J mice. **(B)** Vertebral height of old LG/J was higher than SM/J and C57BL/6 mice. Disc height index (DHI) did not show any difference among the strains at either time-points. **(C-D)** Analysis of reconstructed images from the 3 mouse strains evidenced reduced trabecular bone parameters in LG/J and SM/J compared to C57BL/6 at 23-months, with decrease in BV/TV, trabecular thickness, and number with an increase in trabecular spacing. **(E-F)** Cortical bone analysis showed higher bone volume, cortical thickness, closed porosity and mean polar inertia in LG/J mice compared to both C57BL/6 and SM/J. SM/J presented a decrease in cross sectional thickness along with an increase in closed porosity compared to C57BL/6 and LG/J mice. ANOVA was used to compare normally distributed data, Kruskal-Wallis was for non-normally distributed data, NS = not significant; $p \leq 0.05$ *; $p \leq 0.01$ **; $p \leq 0.01$ ***; C57BL/6 (n=10), LG/J (n=14) and SM/J (n=8) mice, 3 caudal levels; Ca5-7/mouse were analyzed.

**Supplemental Figure 2:** (A) μCT pictures of lumbar spine from 3 representative 23 month-old LG/J, BL6 and SM/J mice show lack of intervertebral disc mineralization, entire lumbar spines (6 discs; L1-S1/mouse) from 10 C57BL/6, 14 LG/J and 8 SM/J mice were analyzed. (B) Alizarin Red staining of calcified caudal disc sections showed presence of high levels of free calcium (red signal) in 23-month-old LG/J. There was lack of any pronounced staining in caudal disc of 23-month-old BL6 and SM/J mice, n= 3 mice/strain were analyzed.

**Supplemental Figure 3:** (A) Histological criterion used for Modified Thompson Grading of NP and AF tissues. (B) Representative pictures showing spectrum of degenerative grades observed in 23-month-old C57BL/6, LG/J and SM/J. (C) Representative picture showing areas selected to assess staining % area for immunohistochemical quantification.

**Supplemental Figure 4: Representative common DEGs that change with aging independent of strain background** (A-B) Representative DEGs from selected GO processes from the common up-regulated DEGs between LG/J 23-month-old vs 6-month-old and BL6 23 month-old vs 6 month-old. **(C)** Representative DEGs from selected GO processes from the common down regulated DEGs between LG/J 23-month-old vs 6-month-old and BL6 23 month-old vs 6 month-old, FDR $\leq 0.05$.

**Supplemental Figure 5: Representative GO processes and select DEGs that change in BL6 aging (A)** Volcano Plot, showing up- and downregulated DEGs from BL6 23M vs BL6 6M comparison, FDR $\leq 0.05$. **(B)** Representative GO processes of upregulated genes in BL6 23M vs BL6 6M. **(C)** Representative GO processes of downregulated genes in BL6 23M vs BL6 6M. **(D)** Representative DEGs from selected GO processes from up-regulated DEGs between BL6 23M vs 6M. **(E)** Representative DEGs from selected GO processes from down-regulated DEGs between BL6 23M vs 6M. DEGs cut-off FDR $\leq 0.05$. GO process enrichment analysis was performed using PANTHER Overrepresentation Test, GO Ontology database annotation, binomial statistical test with FDR $\leq 0.05$.

**Supplemental Figure 6: Representative GO processes and select DEGs that change in LG/J aging (A)** Volcano Plot, showing up- and downregulated DEGs from LG/J 23M vs LG/J

6M comparison, FDR ≤ 0.05. **(B)** Representative GO processes of upregulated genes in LG/J 23M vs LG/J 6M. **(C)** Representative GO processes of downregulated genes in LG/J 23M vs LG/J 6M. **(D)** Representative DEGs from selected GO processes from up-regulated DEGs between LG/J 23M vs 6M. **(E)** Representative DEGs from selected GO processes from down-regulated DEGs between LG/J 23M vs 6M. DEGs cut-off FDR ≤ 0.05. GO process enrichment analysis was performed using PANTHER Overrepresentation Test, GO Ontology database annotation, binomial statistical test with FDR ≤ 0.05

**Supplemental Figure 7: NP marker expression and PCR validation of select genes from DEGs from BL6 and LG/J aging.** (A) NP tissue from caudal discs of 23-month-old LG/J mice showed comparable staining of CA3 and GLUT1 to BL6. SM/J mice showed significantly reduced staining for CA3 and GLUT1. P21 levels were decreased in 23M LG/J compared to 23M BL6 mice. (B) Caudal discs in 23-month-old SM/J mice showed significantly lower number of NP cells compared to LG/J and BL6. (C) 6M LG/J showed higher levels of CASP6 than 6M BL6; CASP4 and RELA were increased in 23M LG/J compared to 6M LG/J and 23M BL6; BL6 presented higher levels of Bcl2 compared to 6M BL6 and 23M LG/J. (D) There were increased levels of POSTN and BMP6, and decreased levels of ALPL and BGLAP in 23M LG/J compared to 23M BL6 mice. (E) There were increased levels of FGF2 and CCND1, and decreased levels of S100a8 and S100a9 in 23M LG/J compared to 23M BL6 mice. (F) 23M LG/J mice presented an increase in CD14 and CD68, CD163 and CDK4 expression compared to 6M LG/J and 23M BL6 mice, respectively. t-test was used to compare normally distributed data, Mann-Whitney test was for non-normally distributed data, $p \leq 0.05$ *; $p \leq 0.01$ **; n=6 mice/strain were analyzed.

**Supplemental Figure 8: Analysis using DEGs obtain from the Hierarchical clustering shown in Figure 6B.** (A) Representative GO processes of downregulated genes between Cluster 4 (degenerated) and Cluster A (healthy), FDR-P-Val≤0.1. (B) Representative GO processes of downregulated genes between Cluster 5 (degenerated) and Cluster A (healthy), FDR-P-Val≤0.1. (C) Representative GO processes of downregulated genes between Cluster 4 (degenerated) and cluster A (healthy), FDR-P-Val≤0.1. (D) Representative GO processes of upregulated genes between Cluster 2 (degenerated) and Cluster A (healthy), FDR-P-Val≤0.1. (E) Representative GO processes of upregulated genes between Cluster 3 (degenerated) and Cluster A (healthy), FDR-P-Val≤0.1. (F) Representative GO processes of upregulated genes between Cluster 5 (degenerated) and Cluster A (healthy), FDR-P-Val≤0.1. (G) Representative schematic summarizing the major enriched pathways enriched in each degeneration cluster. GO processes enrichment analysis was performed using PANTHER Overrepresentation Test, GO Ontology database annotation, binomial statistical test with FDR ≤ 0.05.
